# Time From HIV Diagnosis to Viral Suppression: Survival Analysis of Statewide Surveillance Data in Alabama, 2012 to 2014

**DOI:** 10.2196/17217

**Published:** 2020-05-22

**Authors:** D Scott Batey, Xueyuan Dong, Richard P Rogers, Anthony Merriweather, Latesha Elopre, Aadia I Rana, H Irene Hall, Michael J Mugavero

**Affiliations:** 1 Department of Social Work University of Alabama at Birmingham Birmingham, AL United States; 2 ICF International Fairfax, VA United States; 3 Division of STD Prevention and Control Alabama Department of Public Health Montgomery, AL United States; 4 Department of Medicine University of Alabama at Birmingham Birmingham, AL United States; 5 Division of HIV/AIDS Prevention Centers for Disease Control and Prevention Atlanta, GA United States

**Keywords:** HIV, public health surveillance, sustained viral suppression

## Abstract

**Background:**

Evaluation of the time from HIV diagnosis to viral suppression (VS) captures the collective effectiveness of HIV prevention and treatment activities in a given locale and provides a more global estimate of how effectively the larger HIV care system is working in a given geographic area or jurisdiction.

**Objective:**

This study aimed to evaluate temporal and geographic variability in VS among persons with newly diagnosed HIV infection in Alabama between 2012 and 2014.

**Methods:**

With data from the National HIV Surveillance System, we evaluated median time from HIV diagnosis to VS (<200 c/mL) overall and stratified by Alabama public health area (PHA) among persons with HIV diagnosed during 2012 to 2014 using the Kaplan-Meier approach.

**Results:**

Among 1979 newly diagnosed persons, 1181 (59.67%) achieved VS within 12 months of diagnosis; 52.6% (353/671) in 2012, 59.5% (377/634) in 2013, and 66.9% (451/674) in 2014. Median time from HIV diagnosis to VS was 8 months: 10 months in 2012, 8 months in 2013, and 6 months in 2014. Across 11 PHAs in Alabama, 12-month VS ranged from 45.8% (130/284) to 84% (26/31), and median time from diagnosis to VS ranged from 5 to 13 months.

**Conclusions:**

Temporal improvement in persons achieving VS following HIV diagnosis statewide in Alabama is encouraging. However, considerable geographic variability warrants further evaluation to inform public health action. Time from HIV diagnosis to VS represents a meaningful indicator that can be incorporated into public health surveillance and programming.

## Introduction

The HIV care continuum (“treatment cascade”) is a unifying framework delineating the successive steps following acquisition of HIV infection needed to achieve optimal individual and population health outcomes [1]. The continuum, beginning with serostatus awareness via HIV testing and culminating in plasma HIV viral suppression (VS, <200 c/mL), has been widely adopted for clinical, public health, advocacy, and policy purposes. Indeed, six of the 10 targeted outcomes in the updated National HIV Prevention Indicators for the United States [2] represent discrete steps along the continuum. Individual-level goals focus on attaining higher levels of VS (80% among persons with diagnosed HIV) through increased diagnosis, linkage, and retention in HIV care. A population health-level goal is to reduce new HIV diagnoses by 25%. Similarly, the Joint United Nations Programme on HIV/AIDS has put forth global “90-90-90” targets for three distinct steps on the HIV care continuum: 90% serostatus awareness, 90% antiretroviral therapy (ART) receipt among those with diagnosed HIV, and 90% VS among those receiving ART [3].

Although the value of delineating performance at the successive steps on the continuum is clear, there is an opportunity to take a broader view evaluating success traversing the anchoring steps on the continuum, HIV diagnosis, and VS. Indeed, as HIV surveillance data reported to public health departments and the US Centers for Disease Control and Prevention (CDC) now include reporting of individual-level plasma HIV viral load (VL) values in most jurisdictions in addition to reporting of diagnoses, there is an opportunity to use surveillance data to evaluate VS among persons with newly diagnosed HIV. To this end, we published on a novel HIV surveillance indicator, time from HIV diagnosis to the initial report of VS (<200 c/mL) using publicly reported HIV surveillance data from 19 jurisdictions with comprehensive plasma VL reporting in 2009 [4]. In this study, we observed a median time of 19 months from HIV diagnosis to VS among 17,028 diagnosed persons across jurisdictions. Notably, linkage to care within 3 months of diagnosis (hazard ratio, HR 4.84, 95% CI 4.27-5.48) and better retention in care, as indicated by a higher number of time-updated care visits (HR 1.51 per additional visit, 95% CI 1.48-1.52), were associated with more expeditious VS. From a clinical and public health perspective, a shorter time from HIV diagnosis to VS translates to a reduction in morbidity and mortality and to a reduction in time during which an individual is viremic and likely to transmit HIV [5,6]. People living with HIV who take HIV medicine as prescribed and get and keep an undetectable VL have effectively no risk of transmitting HIV to their HIV-negative sexual partners [7,8]. Similarly, decreasing time between HIV diagnosis and VS and support for the maintenance of VS corresponds to a decrease of circulating virus in the population that can ultimately reduce HIV incidence [9].

Supportive services (eg, case management and transportation assistance), such as those provided through the Ryan White HIV/AIDS Program, are vital for helping shepherd people living with HIV (PLWH) through the HIV care continuum and attaining VS [10]. Similarly, enhanced personal contacts (eg, personalized reminder calls for upcoming appointments and check-ins after missed appointments) increases retention in care [11]. However, evaluation of the time from diagnosis to VS captures the collective effectiveness of HIV prevention and treatment activities in a given locale, including testing, clinical, ART, and supportive services provided by public health, community-based organizations (CBOs), and clinical entities to move persons across the steps of the HIV care continuum [10]. As such, it provides a more global estimate of how effectively the larger HIV care system is working in a given geographic area or jurisdiction and serves a complimentary role to evaluating individual steps on the continuum. In particular, evaluation of temporal and geographic variability in median time from diagnosis to VS may serve as a powerful public health indicator to measure changes over time in response to HIV treatment and prevention initiatives and, more so, identify areas in need of process improvements and/or additional resources. Here, we use data from National HIV Surveillance System (NHSS) to evaluate temporal and geographic variability between 2012 and 2014 across the 11 public health areas (PHAs) in Alabama as a case study of the utility of this novel HIV surveillance indicator to inform public health practice and policy.

## Methods

**Figure 1:**
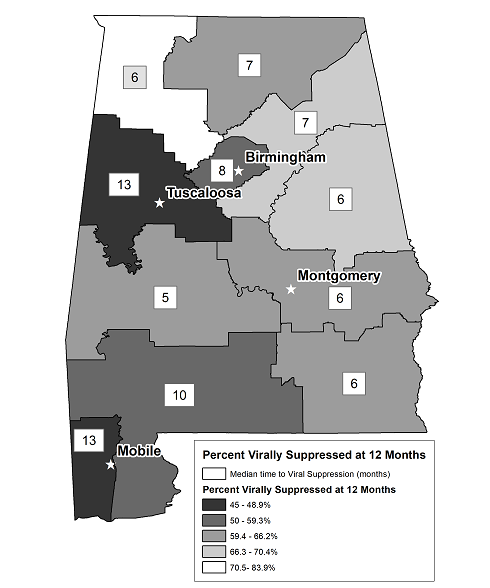
Viral suppression (<200 c/mL) within 12 months of HIV diagnosis and median time to suppression among 1,979 persons with newly diagnosed HIV in Alabama, by Alabama Public Health Area (PHA), 2012-2014.

Historically, Alabama is divided into 11 PHAs (Figure 1), with statewide coordinated HIV prevention and treatment activities led by the Alabama Department of Public Health (ADPH) in conjunction with local health departments, CBOs, and clinical agencies, with HIV care largely supported by the Health Resources and Services Administration via Ryan White funding [12]. Our primary objective was to evaluate temporal and geographic variability in median time from HIV diagnosis to VS by PHA to inform public health action. The ADPH reports cases of HIV, including demographic, clinical, and risk characteristics, to CDC’s NHSS. Reporting was expanded by law in 2011 to include HIV VL test results. All labs in Alabama are required by state law to report diagnostic tests confirming HIV diagnoses and all VL results, including undetectable VLs, to the ADPH. In addition, community and clinical agencies providing HIV testing services are required to submit case report forms, including sociodemographic data, to the ADPH for persons with newly diagnosed HIV to allow for monitoring of epidemiological trends over time. Trained ADPH staff are responsible for follow-up with community and clinical agencies when there is incomplete data reporting on new HIV cases, in many instances extracting the requisite data from agency medical records to ensure complete data capture. The ADPH transmits statewide HIV surveillance data to the CDC without personal identifiers.

For these analyses, we used Alabama statewide HIV surveillance data for calendar years 2012 to 2014 reported to CDC through June 2017 on persons with newly diagnosed HIV aged 13 years or older at diagnosis and residing in Alabama. Vital status of patients and VL test results received from diagnosis till December 2015 were used in the analysis. Analyses are presented by age, sex at birth, race/ethnicity, HIV transmission category (male-to-male sexual contact [men who have sex with men, MSM], injection drug use [IDU], both MSM and IDU, heterosexual contact, or other transmission category), HIV stage at diagnosis (stage 3 [AIDS] or not stage 3) [13], year of diagnosis, PHA of diagnosis as determined by resident county at time of diagnosis, and facility where diagnosis occurred. Descriptive statistics were used to describe the study population as well as the number and proportion of persons achieving VS (<200 c/mL) within 12 months of diagnosis according to sociodemographic, temporal (median times to VS), and geographic variables. Kaplan-Meier approach was used to evaluate proportion without VS and time from HIV diagnosis to VS, defined as the first date with a VL value <200 c/mL. All analyses were conducted using SAS software, version 9.3 (SAS Institute) [14].

As the data for this study was void of personal identifiers and analysis conducted by members of the study team at the CDC in a way that participants cannot be identified, review by an institutional review board was not required.

## Results

Among 1979 persons with HIV infection newly diagnosed in Alabama during 2012 to 2014, most were male (1573/1979, 79.48%), black/African American (1382/1979, 69.83%), aged 20 to 29 (840/1979, 42.45%) or 30 to 39 years (20.87%, 413/1979), MSM (1077/1979, 54.42%), and were not in stage 3 (1537/1979, 77.68%), indicative of less-advanced infection [15] (Table 1). Three PHAs collectively accounted for almost 60% of the new HIV cases—PHA04 (511/1979, 25.82%), PHA08 (369/1979, 18.65%), and PHA11 (284/1979, 14.35%)—which include the urban centers of Birmingham, Montgomery, and Mobile, respectively.

Overall, 1181 persons (1181/1979, 59.68%) achieved VS (<200 c/mL) within 12 months of HIV diagnosis. A higher percentage of women (253/406, 62.3%), whites (311/472, 65.9%), or PLWH of other race/ethnicity (not black, white, or Hispanic/Latino) (50/70, 71%), persons aged 30 to 39 (261/413, 63.2%) or 50 to 59 years (124/195, 63.6%), and those with stage 3 disease (311/442, 70.4%) achieved VS within 12 months of HIV diagnosis (Table 1). Notably, 52.6% (353/671) of persons with HIV diagnosed in 2012 achieved VS within 12 months, whereas 59.5% (377/634) of those with HIV diagnosed in 2013 and 66.9% (451/674) of those with HIV diagnosed in 2014 achieved this biomarker of HIV treatment success. Considerable geographic variability was observed; cross-sectional 12-month VS had a range of 45.8% (130/284) to 84% (26/31) across PHAs in the state (Figure 1).

**Figure 2:**
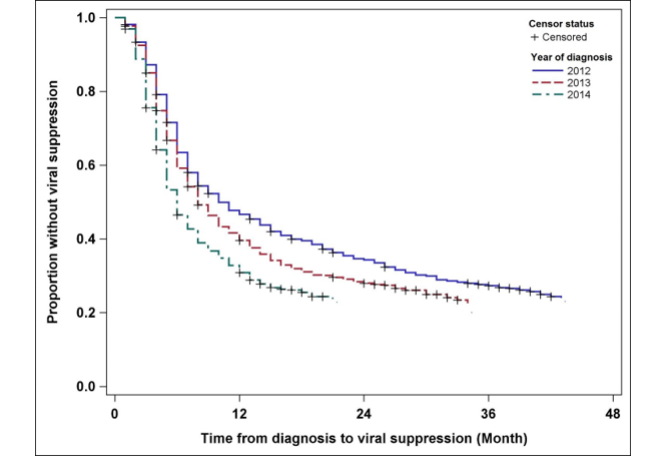
Kaplan-Meier plots of time from HIV diagnosis date to reported first viral suppression (VS, <200 c/mL) among 1979 persons with newly diagnosed HIV ≥13-years-old in Alabama, 2012-2014, stratified by year of diagnosis.

**Figure 3:**
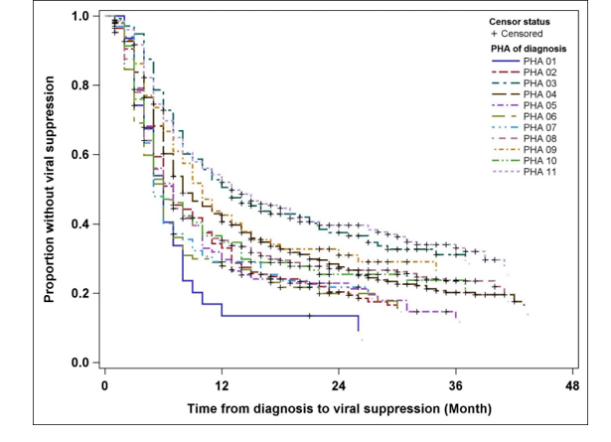
Kaplan-Meier plots of time from HIV diagnosis date to reported first viral suppression (VS, <200 c/mL) among 1979 persons with newly diagnosed HIV ≥13-years-old in Alabama, 2012-2014, stratified by Public Health Area (PHA) of diagnosis.

Among persons with HIV infection diagnosed in Alabama between 2012 and 2014, the median time to achieve VS (<200 c/mL) was eight months. Shorter median time to VS was seen in women, whites, and those identified as other race/ethnicity (not black, white, or Hispanic/Latino), persons over the age of 30 years, those with HIV attributed to IDU or heterosexual contact, and persons with stage 3 disease (all groups ≤7 months; Table 1). Compared with persons with HIV diagnosed in 2012 who achieved VS in a median of 10 months, those with HIV diagnosed in 2013 required a median of eight months, and those diagnosed in 2014 required a median of six months to achieve this HIV biomarker (Figure 2). Considerable heterogeneity was observed in the median time from HIV diagnosis to VS across Alabama’s PHAs, with the exception of PHA03 and PHA11 (Table 1 and Figure 3). The median time of 13 months in these 2 PHAs is considerably higher than 5 to 8 months in the other nine PHAs. PHA03 (including the Tuscaloosa metropolitan statistical area, MSA) and PHA11 (including the Mobile MSA) include more populous regions of the state. In contrast, PHA07, which includes a mostly rural, less-resourced area within Alabama’s Black Belt, had the shortest median time from HIV diagnosis to VS, that is, five months.

**Table 1:** Viral suppression among 1979 persons with newly diagnosed HIV aged 13 years and older in Alabama, 2012 to 2014.

Characteristic				Total, n (%)		VS^a^ within 12 months, n (%)		Median time to VS (95% CI), months^b^
Overall				1979 (100)		1181 (59.7)		8 (7-8)
**Sex**								
	Male		1573 (79.5)		928 (59.0)		8 (8-9)	
	Female		406 (20.5)		253 (62.3)		7 (6-8)	
**Race**								
	Hispanic/Latino		55 (2.8)		31 (56.4)		11 (5-14)	
	Black		1382 (69.8)		789 (57.1)		9 (8-10)	
	White		472 (23.9)		311 (65.9)		7 (6-7)	
	Other		70 (3.5)		50 (71.4)		6 (5-8)	
**Age at diagnosis (years)**								
	13-19		120 (6.1)		69 (57.5)		9 (7-13)	
	20-29		840 (42.4)		486 (57.9)		9 (8-10)	
	30-39		413 (20.9)		261 (63.2)		7 (6-8)	
	40-49		318 (16.1)		195 (61.3)		7 (6-9)	
	50-59		195 (9.9)		124 (63.6)		7 (6-8)	
	60+		93 (4.7)		46 (49.5)		7 (5-13)	
**Transmission category**								
	Male-to-male sexual contact		1077 (54.4)		671 (62.3)		8 (7-9)	
	Injection drug use (IDU)		38 (1.9)		25 (65.8)		7 (5-16)	
	Male-to-male sexual contact and IDU		24 (1.2)		10 (41.7)		16.5 (7^c^)	
	Heterosexual contact		232 (11.7)		145 (62.5)		7 (6-9)	
	Other		608 (30.7)		330 (54.3)		8 (7-10)	
**HIV stage at diagnosis**								
	Not stage 3		1537 (77.7)		870 (56.6)		9 (8-10)	
	Stage 3		442 (22.3)		311 (70.4)		6 (5-6)	
	**Year of diagnosis**							
		2012	671 (33.9)		353 (52.6)		10 (9-13)	
		2013	634 (32.0)		377 (59.5)		8 (8-10)	
		2014	674 (34.1)		451 (66.9)		6 (5-7)	
**Public Health Area (PHA)**								
	PHA01		31 (1.6)		26 (83.9)		6 (4-8)	
	PHA02		177 (8.9)		116 (65.5)		7 (6-9)	
	PHA03		139 (7.0)		68 (48.9)		13 (9-21)	
	PHA04		511 (25.8)		303 (59.3)		8 (7-10)	
	PHA05		98 (5.0)		69 (70.4)		7 (5-9)	
	PHA06		104 (5.3)		73 (70.2)		6 (4-6)	
	PHA07		68 (3.4)		45 (66.2)		5 (5-7)	
	PHA08		369 (18.6)		233 (63.1)		6 (6-7)	
	PHA09		89 (4.5)		49 (55.1)		10 (7-14)	
	PHA10		109 (5.5)		69 (63.3)		6 (5-9)	
	PHA11		284 (14.4)		130 (45.8)		13 (10-19)	

^a^VS: viral suppression.

^b^Median time and 95% CI from diagnosis to the first time of viral suppression during 2012 to 2015.

^c^The upper boundary of the 95% CI for MSM and IDU was missing because its value was beyond the 48 months of the study period from 2012 to 2015.

## Discussion

We observed substantial temporal and geographic variability in VS among persons with HIV infection diagnosed in Alabama between 2012 and 2014. Among 1979 persons, 1181 (1181/1979, 59.67%) achieved VS within 12 months of diagnosis; 52.6% (353/671) in 2012, 59.5% (377/634) in 2013, and 66.9% (451/674) in 2014, with a large decrease in the median time from diagnosis to VS from 10 months to six months, from 2012 to 2014. Considerable geographic variability in 12-month VS and median time to VL suppression was seen across Alabama’s 11 PHAs. Taken together, these observations highlight the considerable heterogeneity and variability in short-term VS, over time and across geographic spaces that include public health areas and geographic regions, among persons with HIV infection diagnosed in Alabama between 2012 and 2014. In the era of rapid ART start programs, time to VS has become a critical indicator of programmatic success. However, it is well noted that sustained VS is essential to maximize individual health outcomes and the population health benefits of U=U (Undetectable=Untransmittable) [16]. As such, cross-sectional 12-month VS gives some indication of sustained VS beyond the initial time to VS metric. However, other methods of measuring sustained VS clearly have value and are needed to best measure longitudinal VL trajectories and maintenance of VS beyond initial success. We suggest that readily available HIV surveillance data, including the novel time from diagnosis to VS indicator, can be used to inform public health action. This can extend to aid in the evaluation of new and ongoing HIV prevention and treatment initiatives in a community, as well as targeted allocation of limited resources to maximize HIV outcomes.

Temporal trends in Alabama from 2012 to 2014 are encouraging, with a four-month decrease (from 10 months to six months) in the median time to achieve VS. Notably, adoption of changes in HIV treatment guidelines recommending universal ART treatment for all persons living with HIV, as well as the increased uptake of integrase strand inhibitors during the observation period, which have more rapid decline in plasma viremia relative to other antiretrovirals, may contribute to the observed large improvement over a relatively short period of time. Our findings provide proof of concept that there is value in monitoring this surveillance indicator to evaluate temporal variability at a population level, as well as to provide a critical variable for modeling exercises evaluating how variability over time (eg, shorter median time to VS following HIV diagnosis) has population-level impact on new HIV infections. Simulation modeling exercises (eg, Markov modeling and agent-based simulations), as espoused by Skarbinski and colleagues [17], could evaluate the impact of varying median times from diagnosis to VS over time and across geographic areas, accounting for disease prevalence, to estimate how shortening the interval to VS would translate to anticipated new HIV cases. As time elapses and more data are available, time from diagnosis to VS could be used in population modeling approaches to evaluate the impact of this interval on observed new HIV cases longitudinally and across geographic areas.

Interestingly, we observed that larger municipalities with likely more resources for HIV prevention, treatment, and supportive services did not necessarily have shorter median times from diagnosis to VS. The broad range of five to 13 months to achieve VS among 1979 persons with diagnosed HIV across Alabama's 11 PHAs is a call to action. To understand this variability, further research is needed within each PHA into the services offered and lived experiences of PLWH, traversing the care continuum from initial diagnosis to VS. We posit that a range of factors at various levels grounded in a socioecological framework, from the individual, interpersonal, community, and health care system, will impact individuals’ trajectories across the continuum, as measured by time from diagnosis to VS [18]. Potentially salient multilevel factors accounting for the variation found among Alabama PHAs may include those associated with suboptimal adherence to ART, such as poverty [19,20] and neighborhood disorder in the community (eg, crime and drug use) [21]. As adherence is an important step in the HIV care continuum and is necessary for achieving VS [22], it is likely that factors which affect adherence also influence time to VS.

Although not the focus of this study, it is also important to consider some of the racial, structural, and geographic factors in Alabama that affect HIV incidence in the state, as these help to contextualize our findings. Black/African Americans are disproportionately affected by HIV in Alabama: although our study found that 69.8% (1382/1979) of new HIV diagnoses in Alabama between 2012 and 2014 were among black/African American people, just over one-quarter (26.8%) of persons living in Alabama identify as black or African American, according to 2018 estimates [23]. Alabama is also one of 14 states to date that has not expanded Medicaid following implementation of the Affordable Care Act [24], thereby creating a coverage gap whereby people with the lowest incomes, below 138% of the Federal Poverty Level, are ineligible for subsidized health insurance through the Marketplace [25]. Lack of Medicaid expansion has negative implications for HIV health, as being uninsured (and without any other health care assistance, as in from the Ryan White HIV/AIDS Program) is associated with increased odds of viral nonsuppression [26]. In addition, as one of the seven states highlighted in the national “Ending the HIV Epidemic” initiative as having a disproportionate incidence of HIV in rural areas [27], Alabama experiences a high HIV burden in rural regions of the state. Although these contextual factors are important for assessing differences in VS across states, they may also help to illuminate some of the intrastate variation in VS found in our study. For example, a possible reason why the mostly black/African American, rural PHAs in Alabama performed better than some of the other, more metropolitan areas of the state may be because of racial segregation, which is common in most Alabama cities. As racial discrimination has been linked to suboptimal ART adherence [28], racial segregation and resultant racial discrimination may help to explain this finding.

As this study exemplifies, it is imperative to gain a better understanding of shared and unique factors across geography to identify the most salient barriers and facilitators, as well as best practices, to emulate toward efforts of expediting the time to VS following HIV diagnosis for all persons, regardless of geography. This oversight would also be applicable and beneficial in other states to inform the generalizability of our findings. Such analyses could provide additional insights on shared and discrepant performance of this HIV surveillance indicator, according to a range of factors grounded in a socioecological framework, which could further inform public health action and resource allocation.

In recent years, increased attention has focused on reducing the time from initial HIV diagnosis to linkage to medical care and ART initiation to achieve better early engagement in HIV medical care and more expeditious VS [29]. Notably, there are often numerous agencies that interact with an individual across the HIV prevention and treatment continua. CBOs and public health departments tend to offer extensive HIV testing as well as other prevention and supportive services. High-impact prevention activities, as defined by the CDC as evidence-based, have expanded in many instances to include linkage to care and ART adherence programs, affecting subsequent steps on the care continuum. Evidence informed activities, such as the Data to Care initiative to use surveillance data to identify out-of-care PLWH and link them to care, are also important for helping PLWH move through steps of the HIV care continuum [30]. On-going attendance and retention in medical care is also needed to optimize sustained ART receipt to achieve VS. Rather than evaluating individual steps along the HIV care continuum, time from diagnosis to VS is a surveillance indicator that captures the successful, expeditious traverse through the care continuum as a result of the collective efforts between numerous agencies. As such, the performance of this indicator may represent the effectiveness of the response and delivery of services within a community or geographic area. However, we suggest these data can provide an objective measure that can be tracked over time to assess, in part, the effectiveness of linkage to care and treatment services affecting the disease locally. The results of several recent trials in urban domestic and international settings have indicated that rapid ART initiation, including starting ART on the same day as HIV diagnosis, shows promise in improving patient and programmatic outcomes, including improved linkage to care, early retention in care, and, indeed, shorter time to VS [29,31,32]. As suggested earlier, a more detailed understanding of an individual’s experience traversing the care continuum within a geographic area, such as within each PHA in Alabama, is essential to inform our interpretation of the widespread variability in VS by place and to guide a more efficient and effective statewide coordinated HIV plan.

Limitations of our study include the potential for underreporting of VL values that could impact the time from HIV diagnosis to VS. However, we note that widespread efforts from the ADPH to monitor laboratory reporting and provide feedback as well as technical assistance would negate impact on study findings. Furthermore, we were only able to observe persons with HIV diagnosed over a three-year period from 2012 to 2014 because of the relatively new implementation of HIV biomarker reporting in our state and the required lags for data reporting. However, temporal improvement was still observed and lends to this surveillance indicator being a useful tool to monitor efficacy of community-level programs. In addition, its application in other states and jurisdictions will allow for more mature and robust reporting through the National HIV Surveillance System. It was beyond the scope of this study to further explore other factors that may have been associated with the geographic heterogeneity seen, including locally coordinated high-impact prevention efforts, barriers to and facilitators of primary medical care access, and the lived experiences of individuals with diagnosed HIV, especially as these are affected by HIV-related stigma. These will be critical areas for future research. As the focus of our study was on temporal and geographic variability, we did not control for sociodemographic differences in assessing VS within 12 months and median time to VS. Future research should account for individual-level variation. In addition, future research should assess whether these differences in VS across Alabama PHAs represent durable patterns or vary over time.

### Public Health Implications

We describe the application of a novel HIV surveillance indicator, time from HIV diagnosis to VS, which is readily captured from data that are reported to state health departments and the CDC. The temporal and geographic variability in this HIV surveillance indicator among persons with HIV diagnosed in Alabama between 2012 and 2014 provides proof of concept of how incorporation of this metric could inform public health practice within jurisdictions, states, and geographic regions in the United States. This novel surveillance indicator, spanning the steps of the HIV care continuum from testing to VS, represents a composite measure of the effectiveness of HIV prevention, treatment, and supportive service provision within a locale and can be used to measure trends over time and across geographic territory. Further research, grounded in a socioecological framework, exploring individual and contextual factors that may contribute to heterogeneity seen in this study, is essential to inform and to guide a tailored public health plan to maximize population health impact.

## Abbreviations

ADPH: Alabama Department of Public Health
ART: antiretroviral therapy
CBOs: community-based organizations
CDC: Centers for Disease Control and Prevention
IDU: injection drug use
MSM: men who have sex with men
NHSS: National HIV Surveillance System
PHA: public health area
PLWH: people living with HIV
VL: viral load
VS: viral suppression

## References

[1] (2017). Centers for Disease Control and Prevention.

[2] (2015). HIV.gov.

[3] UNAIDS (2014). The Joint United Nations Programme on HIV/AIDS (UNAIDS).

[4] Hall HI, Tang T, Westfall AO, Mugavero MJ (2013). HIV care visits and time to viral suppression, 19 US jurisdictions, and implications for treatment, prevention and the national HIV/AIDS strategy. PLoS One.

[5] Quinn TC, Wawer MJ, Sewankambo N, Serwadda D, Li C, Wabwire-Mangen F, Meehan MO, Lutalo T, Gray RH (2000). Viral load and heterosexual transmission of human immunodeficiency virus type 1. Rakai Project Study Group. N Engl J Med.

[6] Cohen M, Chen Y, McCauley M, Gamble T, Hosseinipour M, Kumarasamy N, Hakim J, Kumwenda J, Grinsztejn B, Pilotto J, Godbole S, Mehendale S, Chariyalertsak S, Santos BR, Mayer KH, Hoffman IF, Eshleman SH, Piwowar-Manning E, Wang L, Makhema J, Mills LA, de Bruyn G, Sanne I, Eron J, Gallant J, Havlir D, Swindells S, Ribaudo H, Elharrar V, Burns D, Taha TE, Nielsen-Saines K, Celentano D, Essex M, Fleming TR, HPTN 052 Study Team (2011). Prevention of HIV-1 infection with early antiretroviral therapy. N Engl J Med.

[7] The Lancet Hiv (2017). U=U taking off in 2017. Lancet HIV.

[8] McCray E, Mermin J (2017). Samaritan Ministry.

[9] Montaner JS, Lima VD, Barrios R, Yip B, Wood E, Kerr T, Shannon K, Harrigan PR, Hogg RS, Daly P, Kendall P (2010). Association of highly active antiretroviral therapy coverage, population viral load, and yearly new HIV diagnoses in British Columbia, Canada: a population-based study. Lancet.

[10] Kay ES, Batey DS, Mugavero MJ (2018). The Ryan White HIV/AIDS Program: supplementary service provision post-affordable care act. AIDS Patient Care STDS.

[11] Gardner L, Giordano T, Marks G, Wilson TE, Craw JA, Drainoni ML, Keruly JC, Rodriguez AE, Malitz F, Moore RD, Bradley-Springer LA, Holman S, Rose CE, Girde S, Sullivan M, Metsch LR, Saag M, Mugavero MJ, Retention in Care Study Group (2014). Enhanced personal contact with HIV patients improves retention in primary care: a randomized trial in 6 US HIV clinics. Clin Infect Dis.

[12] Alabama Department of Public Health (ADPH).

[13] (1992). 1993 revised classification system for HIV infection and expanded surveillance case definition for AIDS among adolescents and adults. MMWR Recomm Rep.

[14] SAS (2006). Base SAS 9.1.3 Procedures Guide. Second Edition.

[15] Centers for Disease Control Prevention (CDC) (2014). Revised surveillance case definition for HIV infection--United States, 2014. MMWR Recomm Rep.

[16] Prevention Access Campaign: U=U.

[17] Skarbinski J, Rosenberg E, Paz-Bailey G, Hall HI, Rose CE, Viall AH, Fagan JL, Lansky A, Mermin JH (2015). Human immunodeficiency virus transmission at each step of the care continuum in the United States. JAMA Intern Med.

[18] Mugavero MJ, Amico KR, Horn T, Thompson MA (2013). The state of engagement in HIV care in the United States: from cascade to continuum to control. Clin Infect Dis.

[19] Siefried KJ, Mao L, Kerr S, Cysique LA, Gates TM, McAllister J, Maynard A, de Wit J, Carr A, PAART study investigators (2017). Socioeconomic factors explain suboptimal adherence to antiretroviral therapy among HIV-infected Australian adults with viral suppression. PLoS One.

[20] Kalichman SC, Hernandez D, Kegler C, Cherry C, Kalichman MO, Grebler T (2015). Dimensions of poverty and health outcomes among people living with HIV infection: limited resources and competing needs. J Community Health.

[21] Surratt HL, Kurtz SP, Levi-Minzi MA, Chen M (2015). Environmental influences on HIV medication adherence: the role of neighborhood disorder. Am J Public Health.

[22] Byrd K, Hou J, Bush T, Hazen R, Kirkham H, Delpino A, Weidle PJ, Shankle MD, Camp NM, Suzuki S, Clay PG, Patient-centered HIV Care Model Team (2020). Adherence and viral suppression among participants of the patient-centered human immunodeficiency virus (HIV) care model project: a collaboration between community-based pharmacists and HIV clinical providers. Clin Infect Dis.

[23] United States Census Bureau.

[24] Kaiser Family Foundation.

[25] Garfield R, Orgera K, Damico A (2020). Kaiser Family Foundation.

[26] Bradley H, Viall AH, Wortley PM, Dempsey A, Hauck H, Skarbinski J (2016). Ryan White HIV/AIDS Program Assistance and HIV Treatment Outcomes. Clin Infect Dis.

[27] Fauci AS, Redfield RR, Sigounas G, Weahkee MD, Giroir BP (2019). Ending the HIV epidemic: a plan for the United States. J Am Med Assoc.

[28] Bogart LM, Wagner GJ, Galvan FH, Klein DJ (2010). Longitudinal relationships between antiretroviral treatment adherence and discrimination due to HIV-serostatus, race, and sexual orientation among African-American men with HIV. Ann Behav Med.

[29] Pilcher CD, Ospina-Norvell C, Dasgupta A, Jones D, Hartogensis W, Torres S, Calderon F, Demicco E, Geng E, Gandhi M, Havlir DV, Hatano H (2017). The effect of same-day observed initiation of antiretroviral therapy on HIV viral load and treatment outcomes in a US public health setting. J Acquir Immune Defic Syndr.

[30] Centers for Disease Control and Prevention.

[31] Koenig SP, Dorvil N, Dévieux JG, Hedt-Gauthier BL, Riviere C, Faustin M, Lavoile K, Perodin C, Apollon A, Duverger L, McNairy ML, Hennessey KA, Souroutzidis A, Cremieux P, Severe P, Pape JW (2017). Same-day HIV testing with initiation of antiretroviral therapy versus standard care for persons living with HIV: A randomized unblinded trial. PLoS Med.

[32] Rosen S, Maskew M, Fox MP, Nyoni C, Mongwenyana C, Malete G, Sanne I, Bokaba D, Sauls C, Rohr J, Long L (2016). Initiating antiretroviral therapy for HIV at a patient's first clinic visit: The RapIT Randomized Controlled Trial. PLoS Med.

